# Supplementary material for: Magnetic charge propagation upon a 3D artificial spin-ice
Source: Nat Commun. 2021 May 28;12:3217. doi: 10.1038/s41467-021-23480-7 (PMC8163774; doi:10.1038/s41467-021-23480-7)
Supplement: Supplementary file 1 — Supplementary Information [file 41467_2021_23480_MOESM1_ESM.pdf]

# Supplementary Information

## Magnetic Charge Propagation upon a 3D Artificial Spin-ice

A. May[1], M. Saccone[2], A. van den Berg[1], J. Askey[1], M. Hunt[1] and S. Ladak*[1]

Correspondence to: ladaks@cardiff.ac.uk

**This PDF file includes:**

Figs. S1 to S12

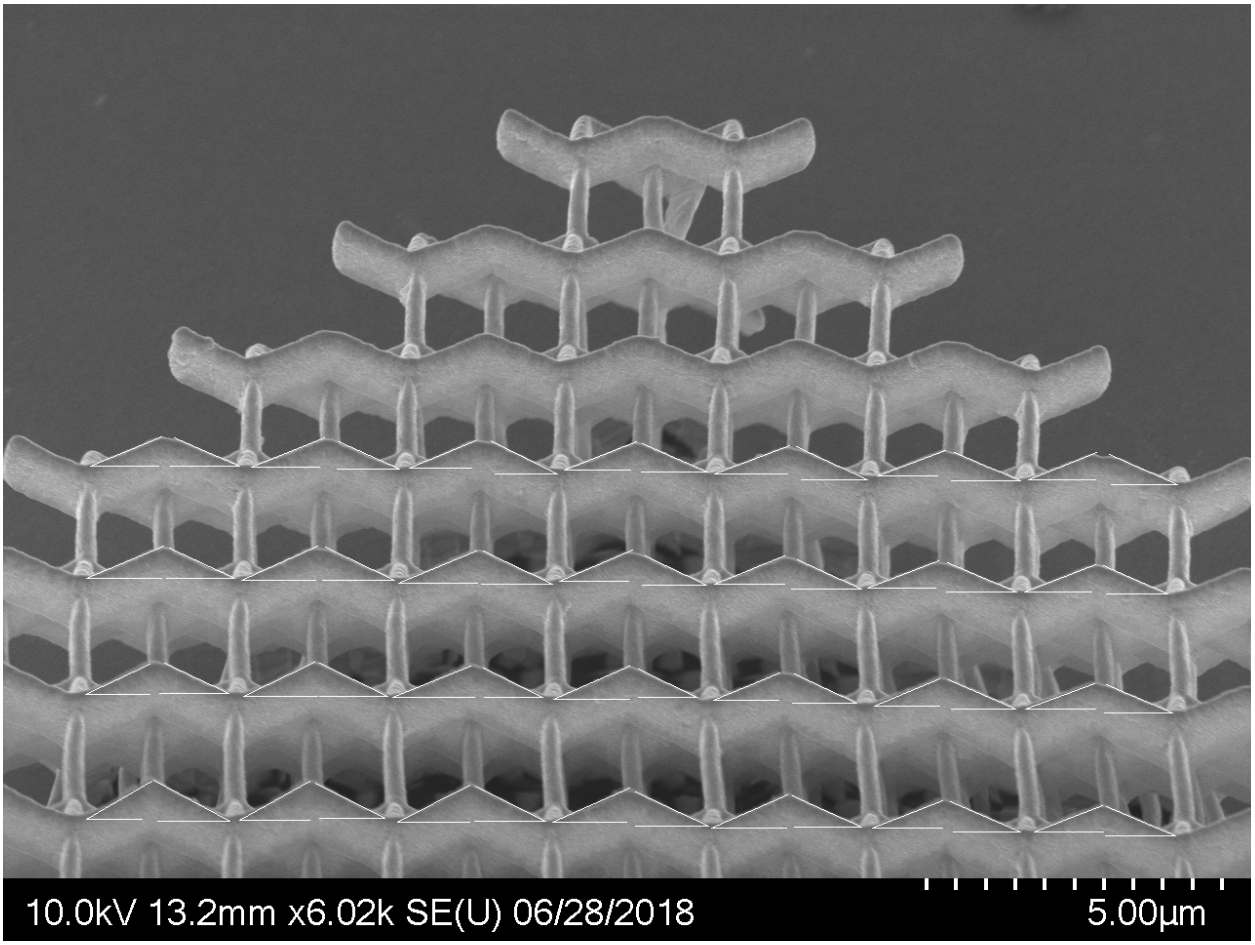


**Supplementary Fig. 1**: SEM image showing 50 measurements of the angle between the L1 wire long-axis and the substrate plane (θ). A simple calculation was required to transform this measured angle into the actual angle, to account for the 45° tilt present during image acquisition.

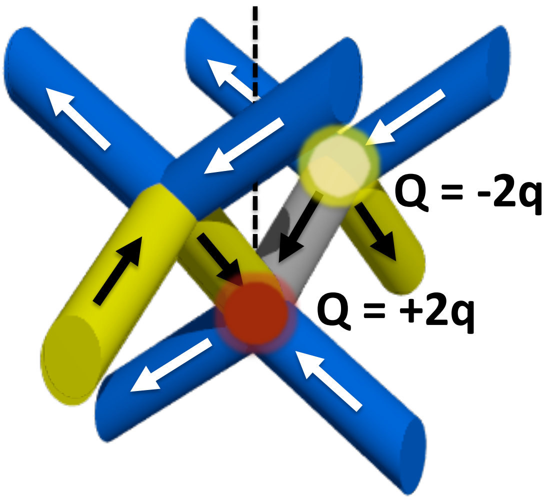


**Supplementary Fig. 2**: A schematic showing the creation of monopoles upon the L2 sub-lattice.

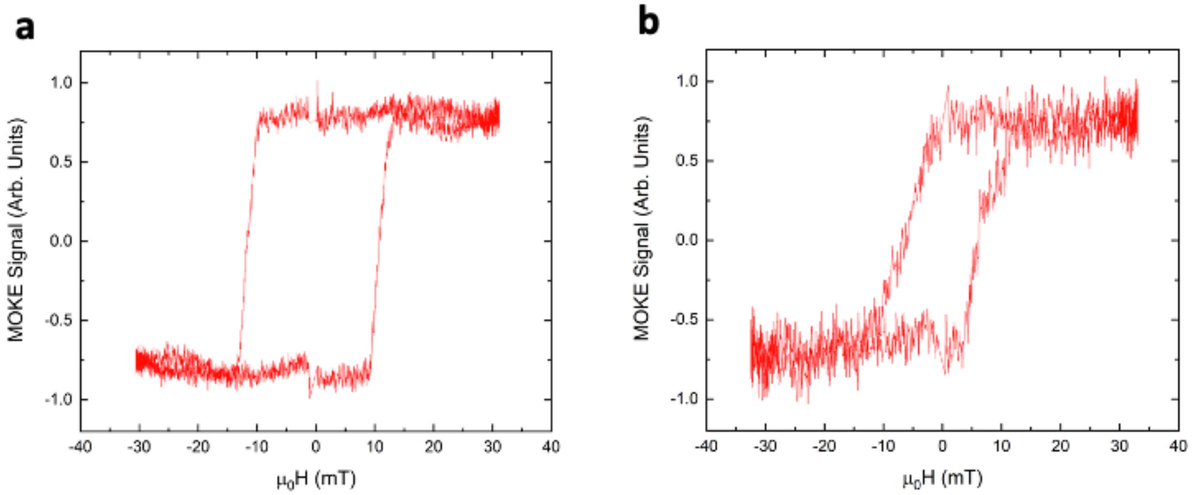


**Supplementary Fig. 3**: Optical magnetometry with external field applied along projection of **(a)** L1 sub-lattice and **(b)** L2 sub-lattice.

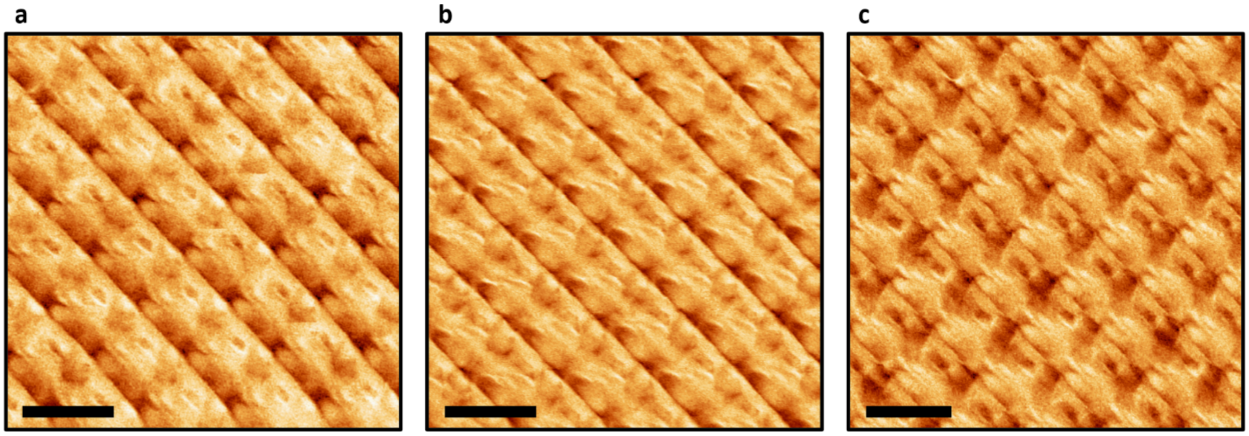


**Supplementary Fig. 4**: Unmasked MFM images of L1 and L2 saturated states. (a) An MFM image taken at remanence after application of saturating fields along the unit vectors (1,-1,0) and (-1,-1,0). (b) MFM image taken at remanence after a further saturating field is now applied along unit vector (-1,1,0). (c) MFM image taken at remanence after a further saturating field is now applied along unit vectors (1,-1,0) and (1,1,0). See main text for definition of coordinate system. Scale bars are 2μm.

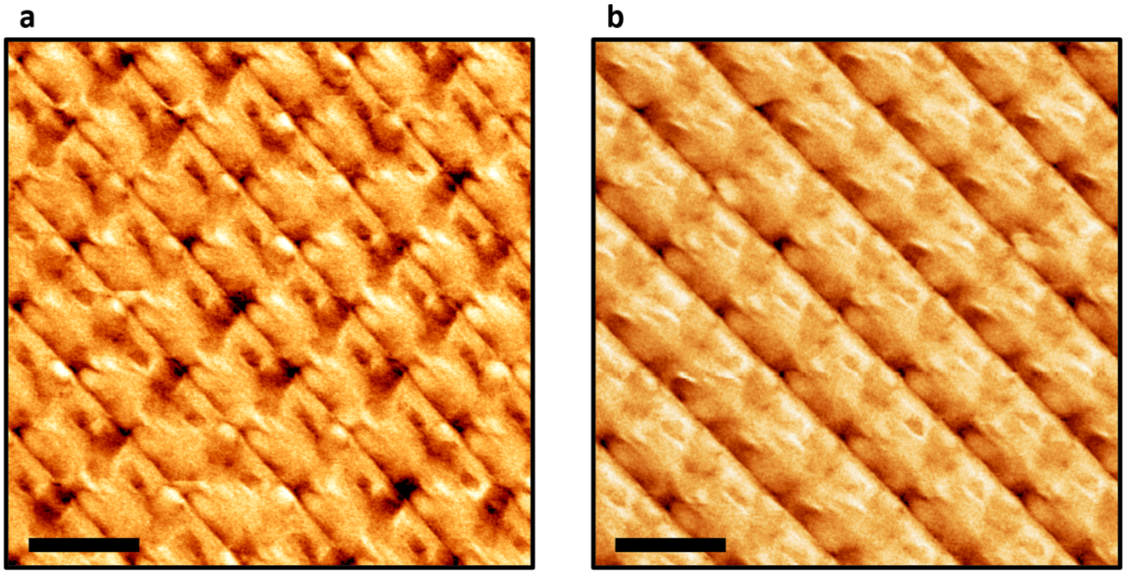


**Supplementary Fig. 5**: Unmasked MFM images of intermediate L1 and L2 states. (a) MFM image taken at remanence following a saturating field along L2, unit vector (-1,-1,0), and subsequent 8.0mT field applied along (1,1,0). (b) MFM image taken at remanence following a saturating field along L1, the unit vector (1,-1,0), and subsequent 9.5mT field along the unit vector (-1,1,0). See main text for definition of coordinate system.

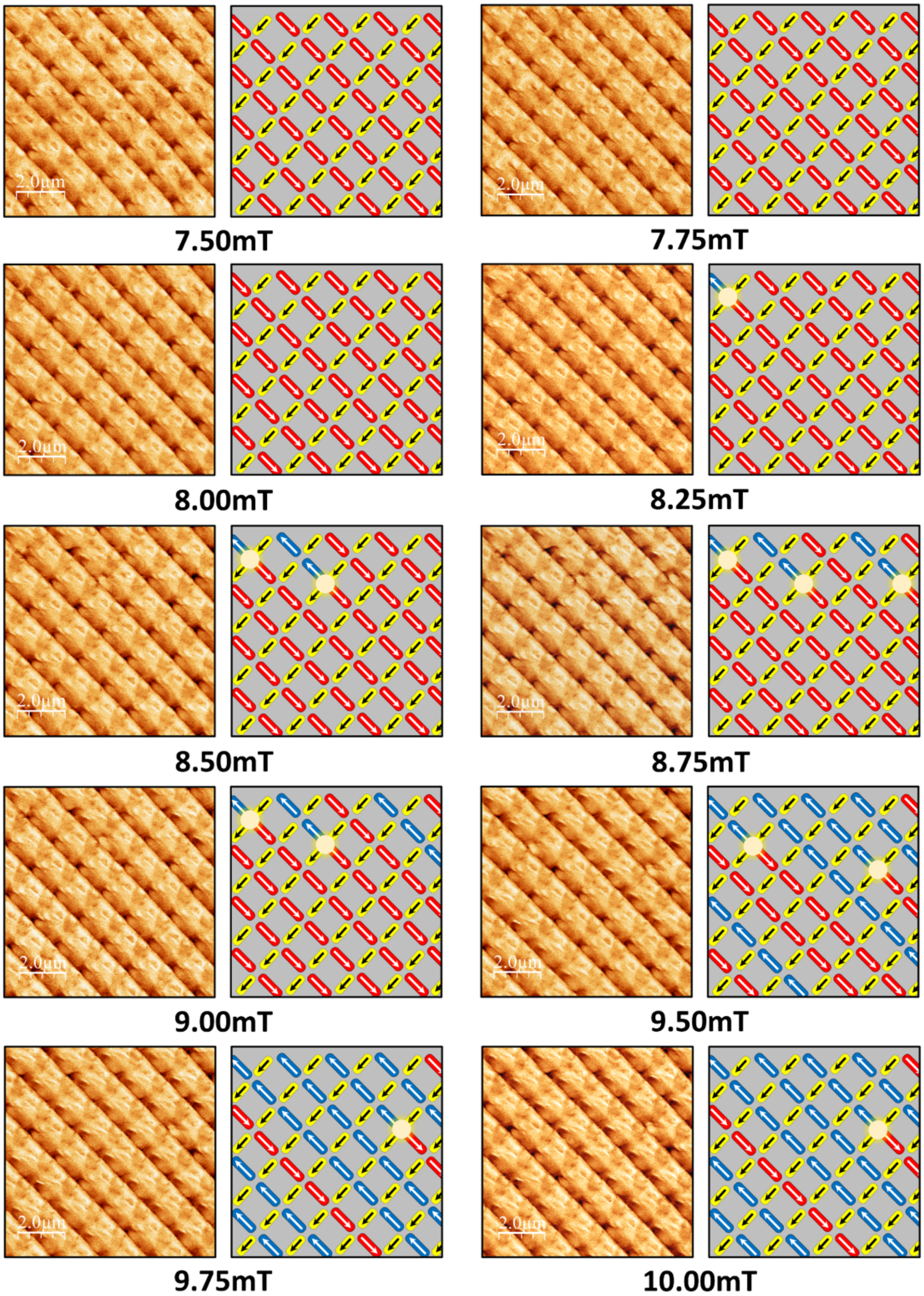


**Supplementary Fig. 6**: Raw MFM data without masks, L1 switching. All scale bars are 2μm.

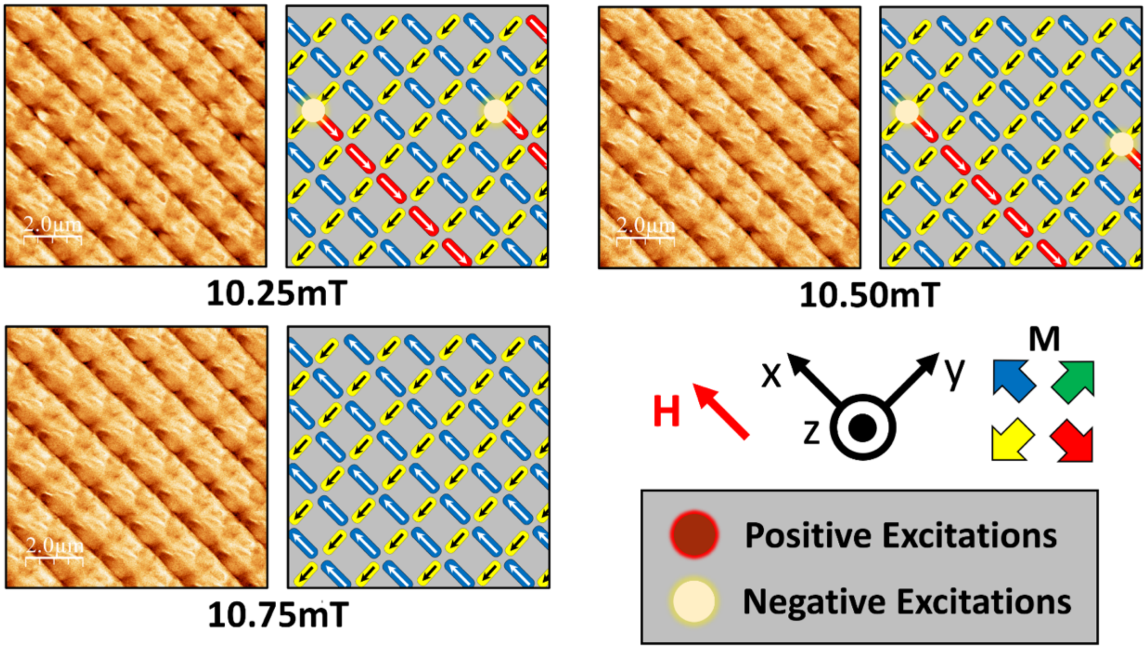


**Supplementary Fig. 6 (continued):** Raw MFM data without masks, L1 switching. All scale bars are 2μm.

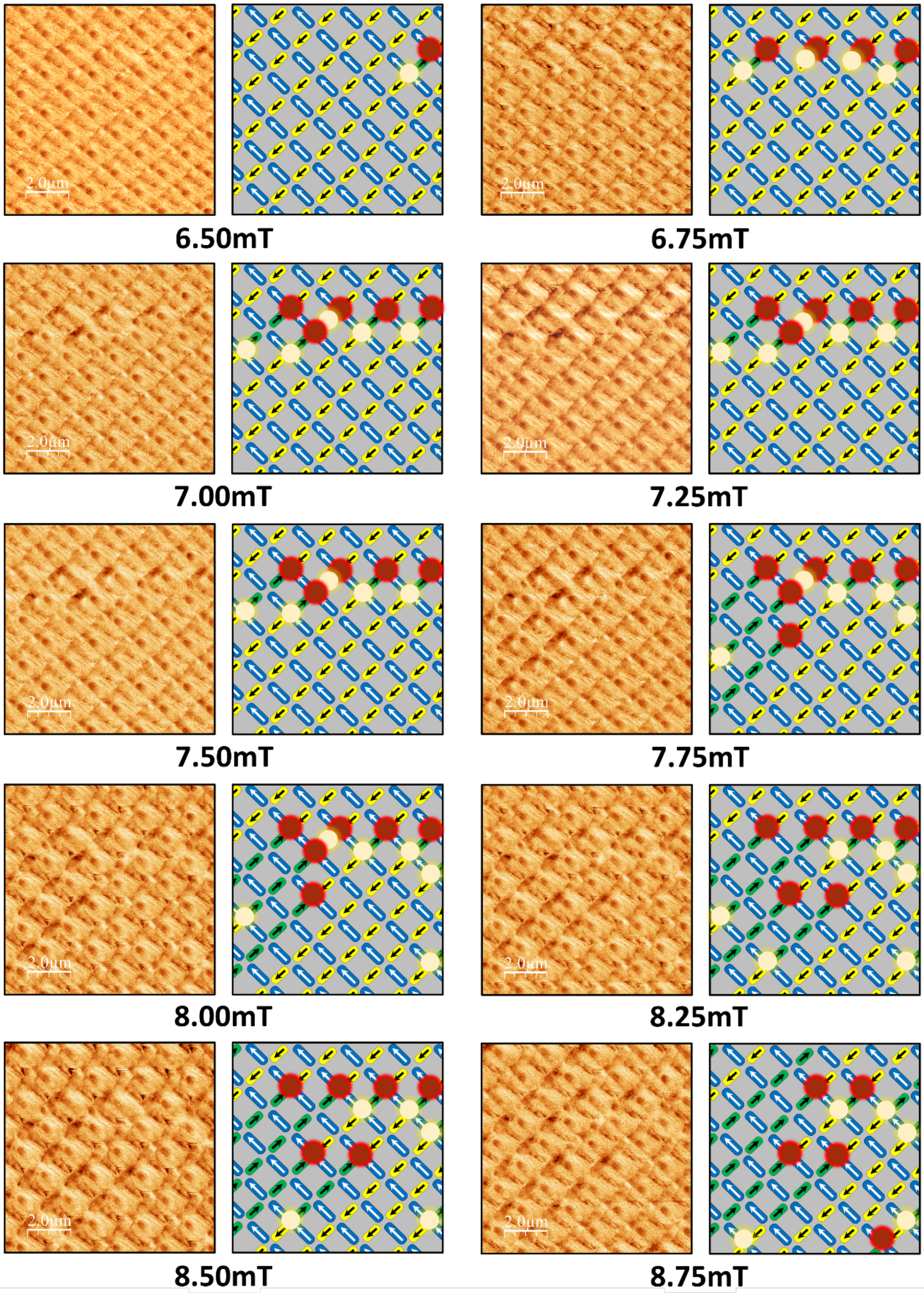


**Supplementary Fig. 7:** Raw MFM data without masks, L2 switching. All scale bars are 2µm.

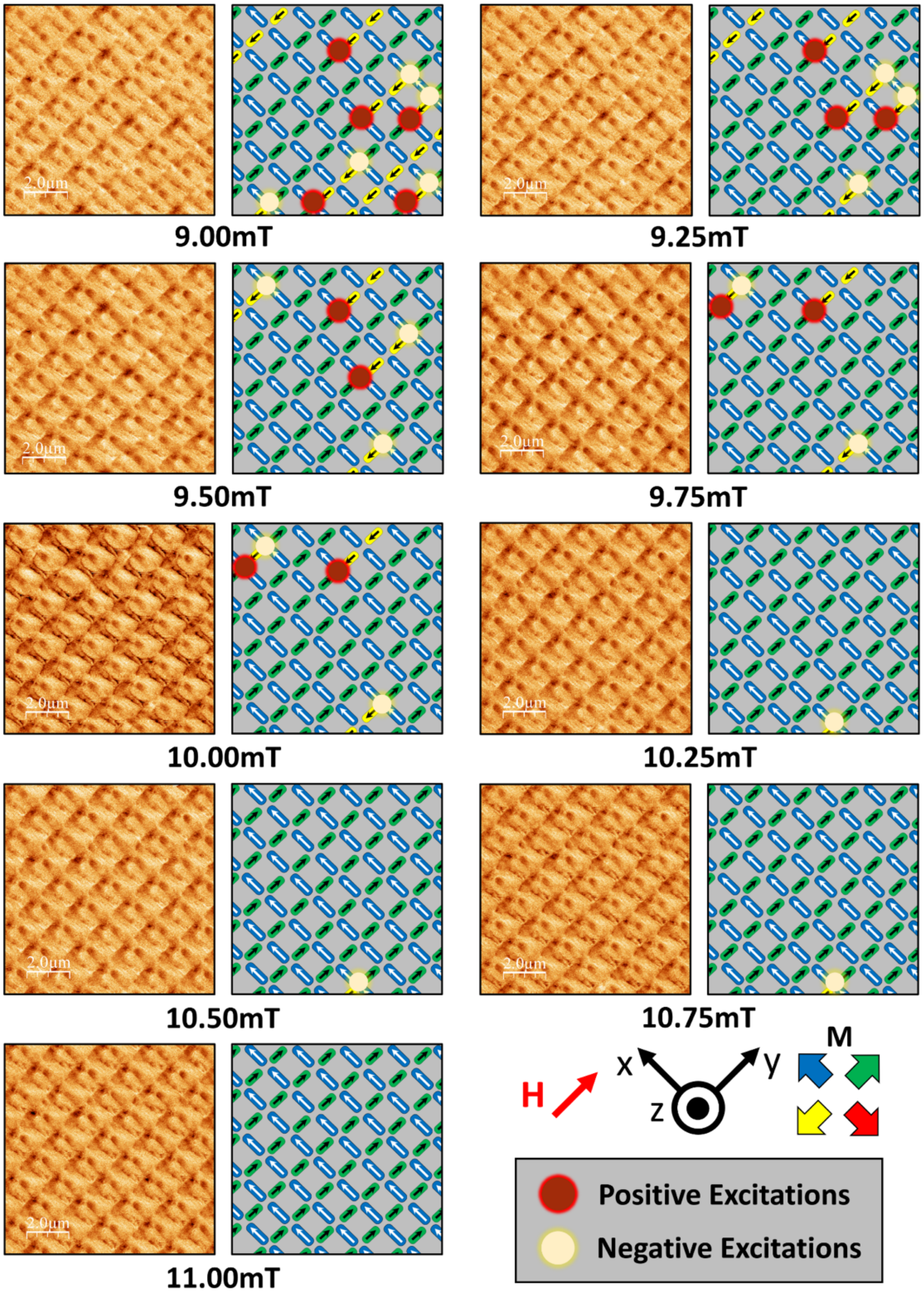


**Supplementary Fig. 7 (continued):** Raw MFM data without masks, L2 switching. All scale bars are 2μm.

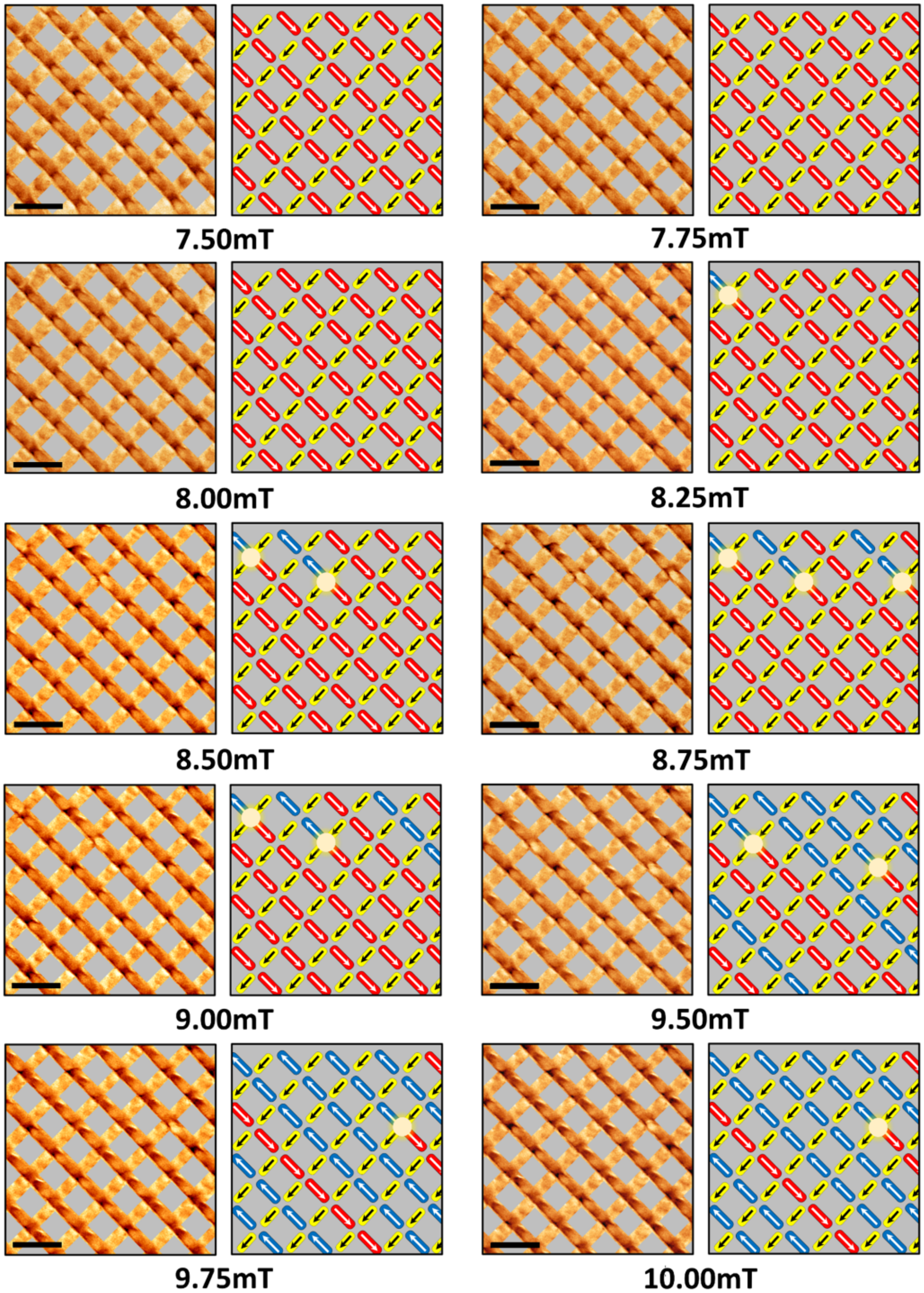


**Supplementary Fig. 8**: Full MFM data with masks, L1 switching. All scale bars are 2μm.

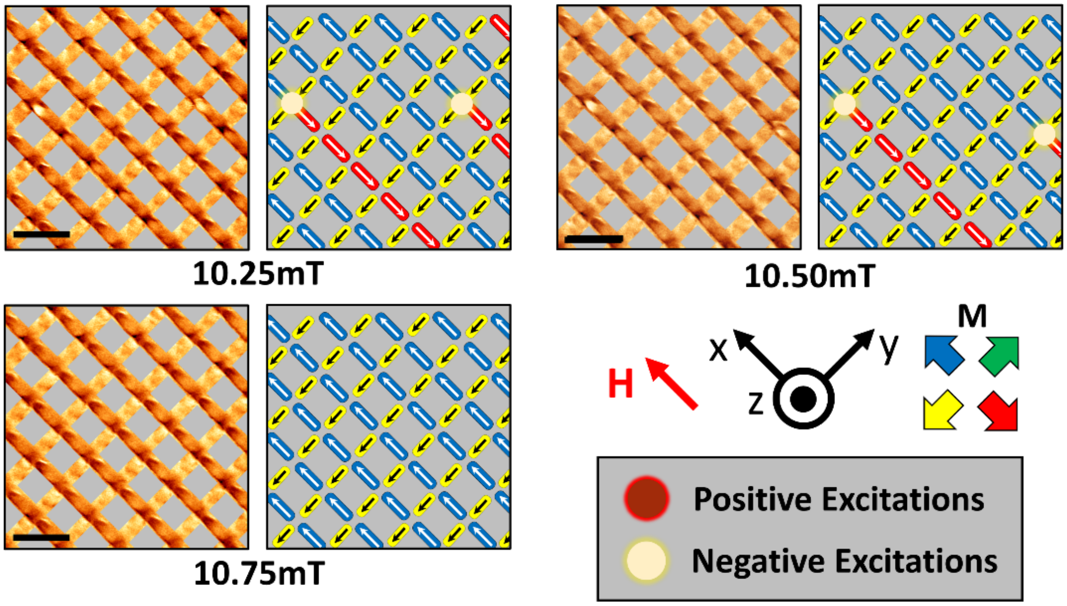


**Supplementary Fig. 8 (continued)**: Full MFM data with masks, L1 switching. All scale bars are 2µm.

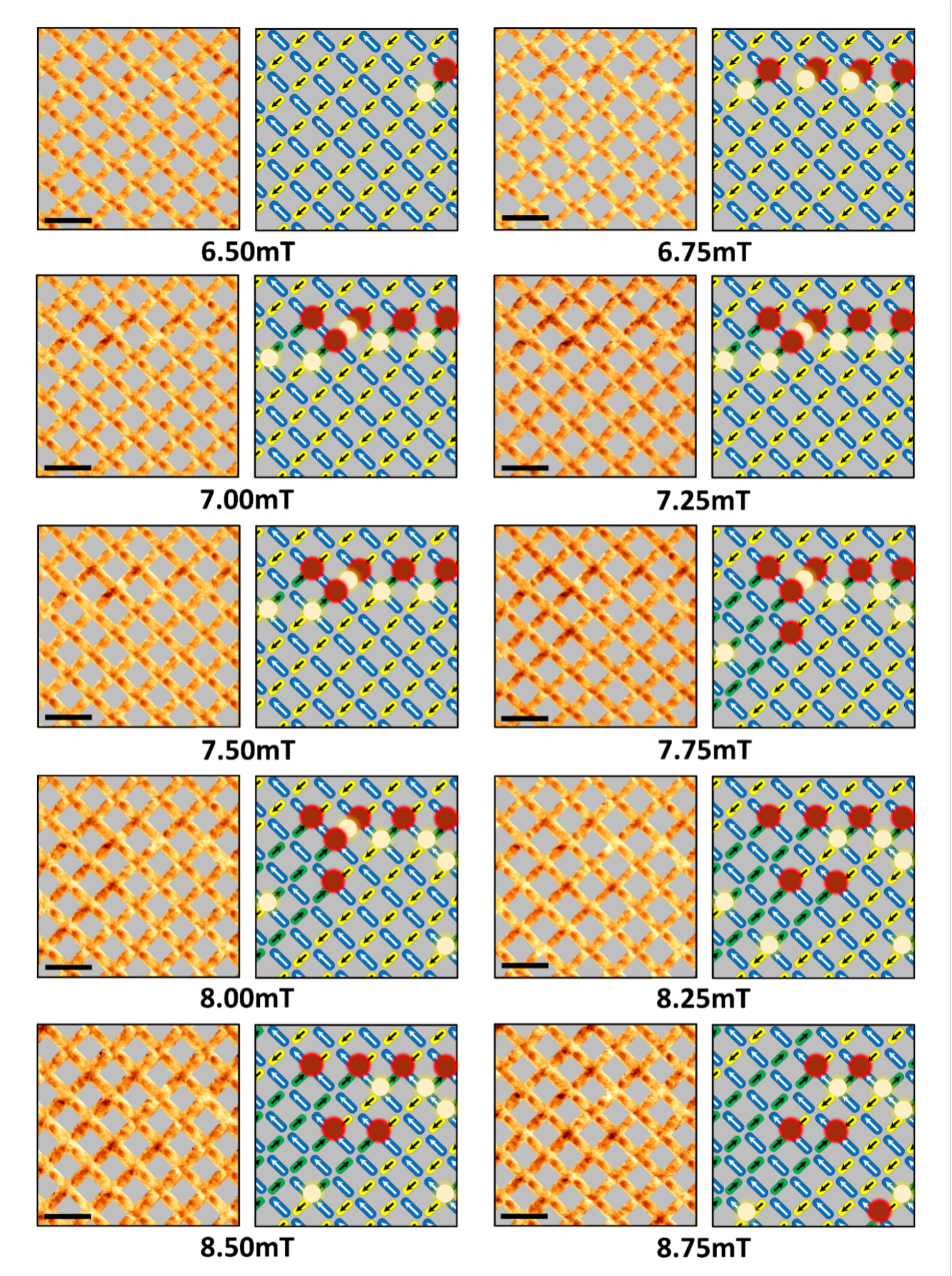


**Supplementary Fig. 9:** Full MFM data with masks, L2 switching. All scale bars are 2μm.

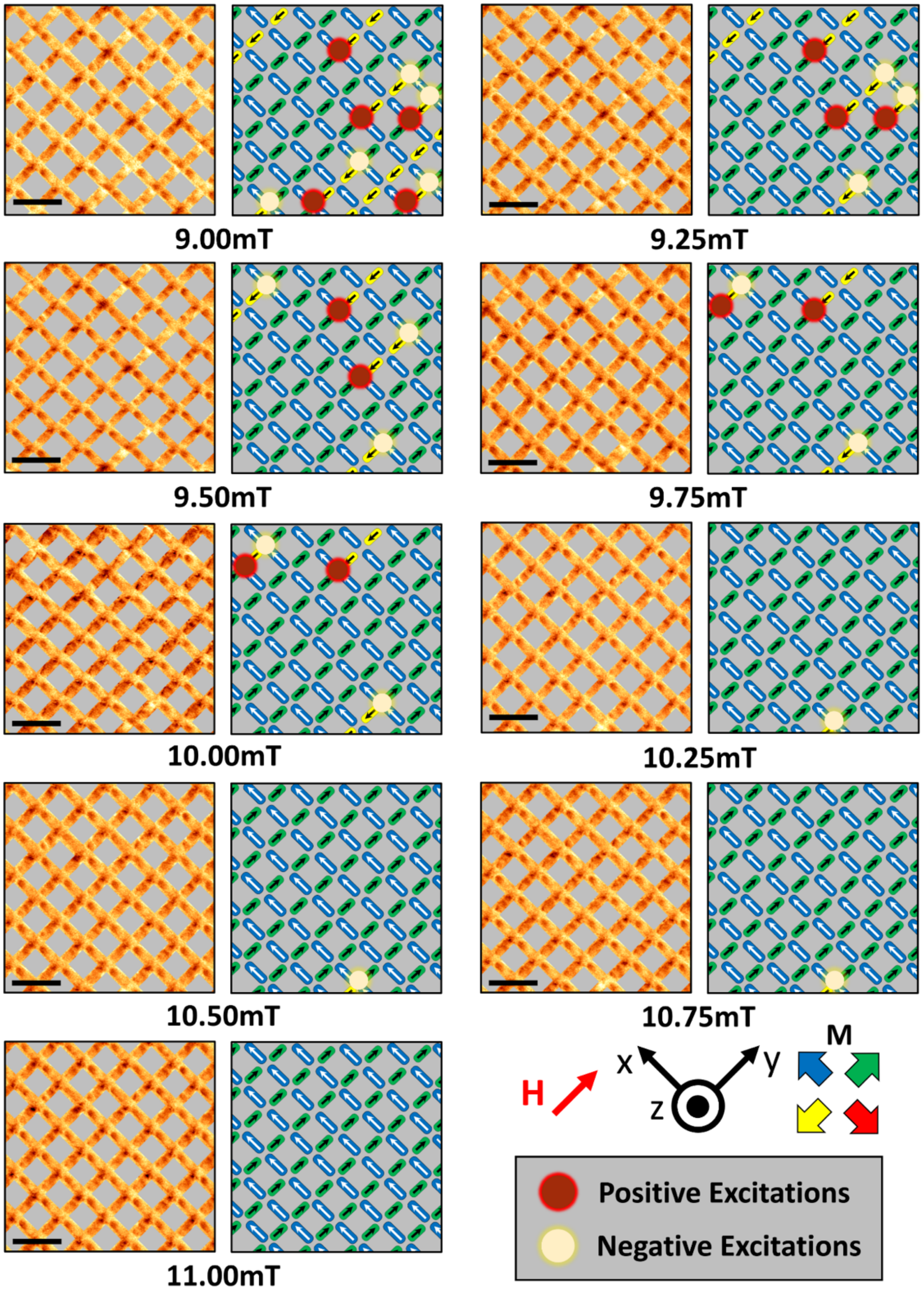


**Supplementary Fig. 9 (continued)**: Full MFM data with masks, L2 switching. All scale bars are 2μm.

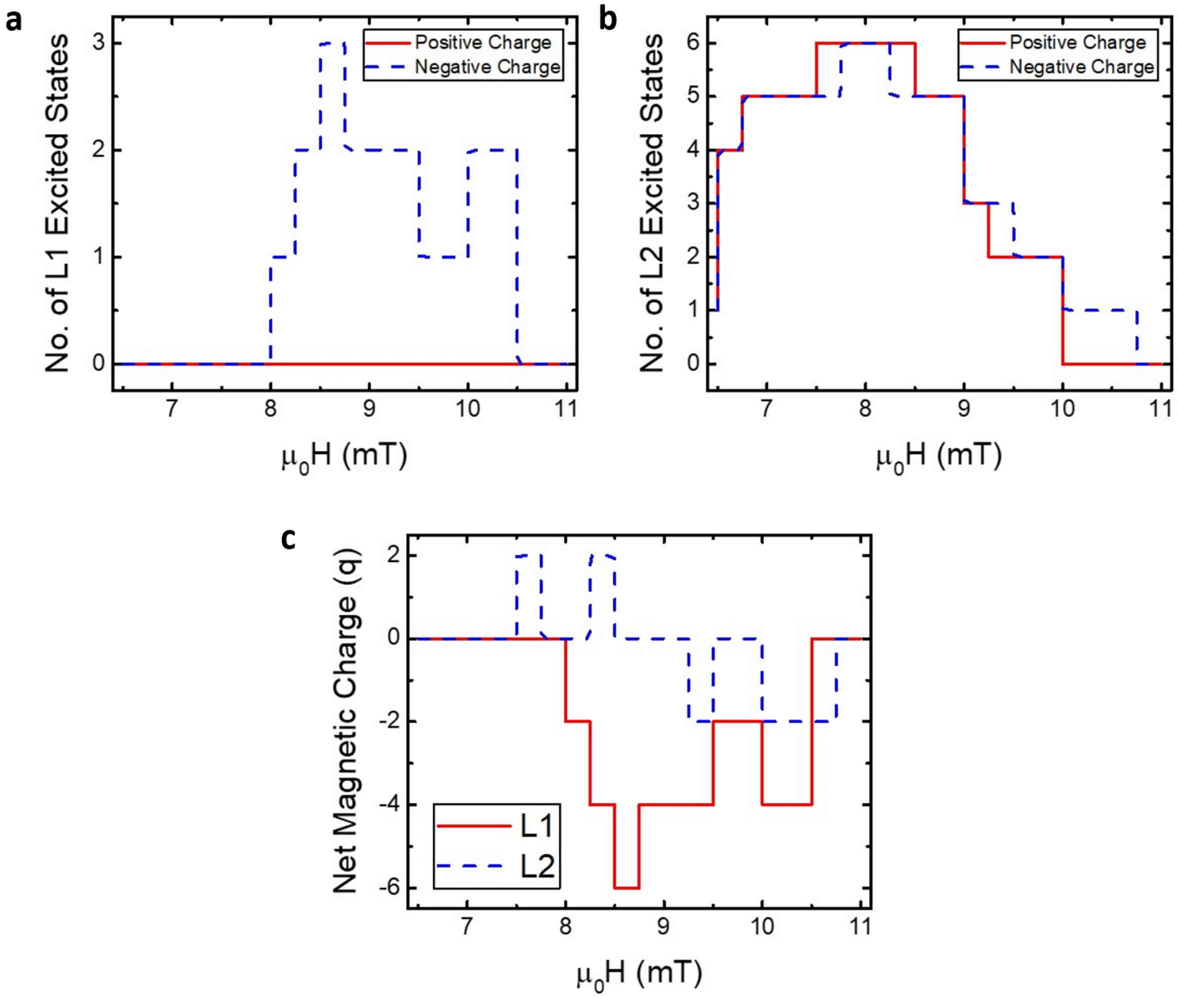


**Supplementary Fig. 10:** Analysis of MFM reversal datasets. **(a)** Number of monopole-excitations as a function of applied field for the L1 switching. Excitations are separated into Q = +2q (red) and Q = -2q (blue). **(b)** Equivalent plot for observed monopole-excitations during the L2 reversal. **(c)** Net magnetic charge within the measured window during the L1 (red) and L2 (blue) reversals.

| Vertex Diagram | Vertex Type | Energy Density ($Jm^{-3}$) |
|---|---|---|
| 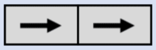 | 1-in/1-out | 7021 |
| 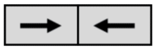 | 2-in | 16006 |
| 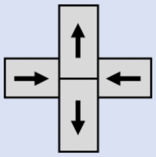 | 1 | 8342 |
| 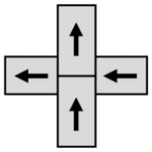 | 2 | 7745 |
| 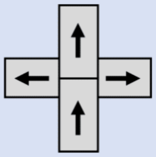 | 3 | 9907 |
| 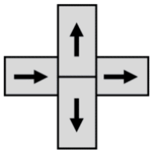 | 3 | 11276 |
| 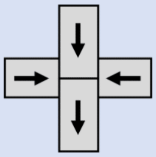 | 3 | 10447 |
| 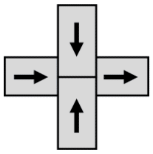 | 3 | 11683 |

**Supplementary Fig. 11**: Vertex energies as calculated using micromagnetic simulations.

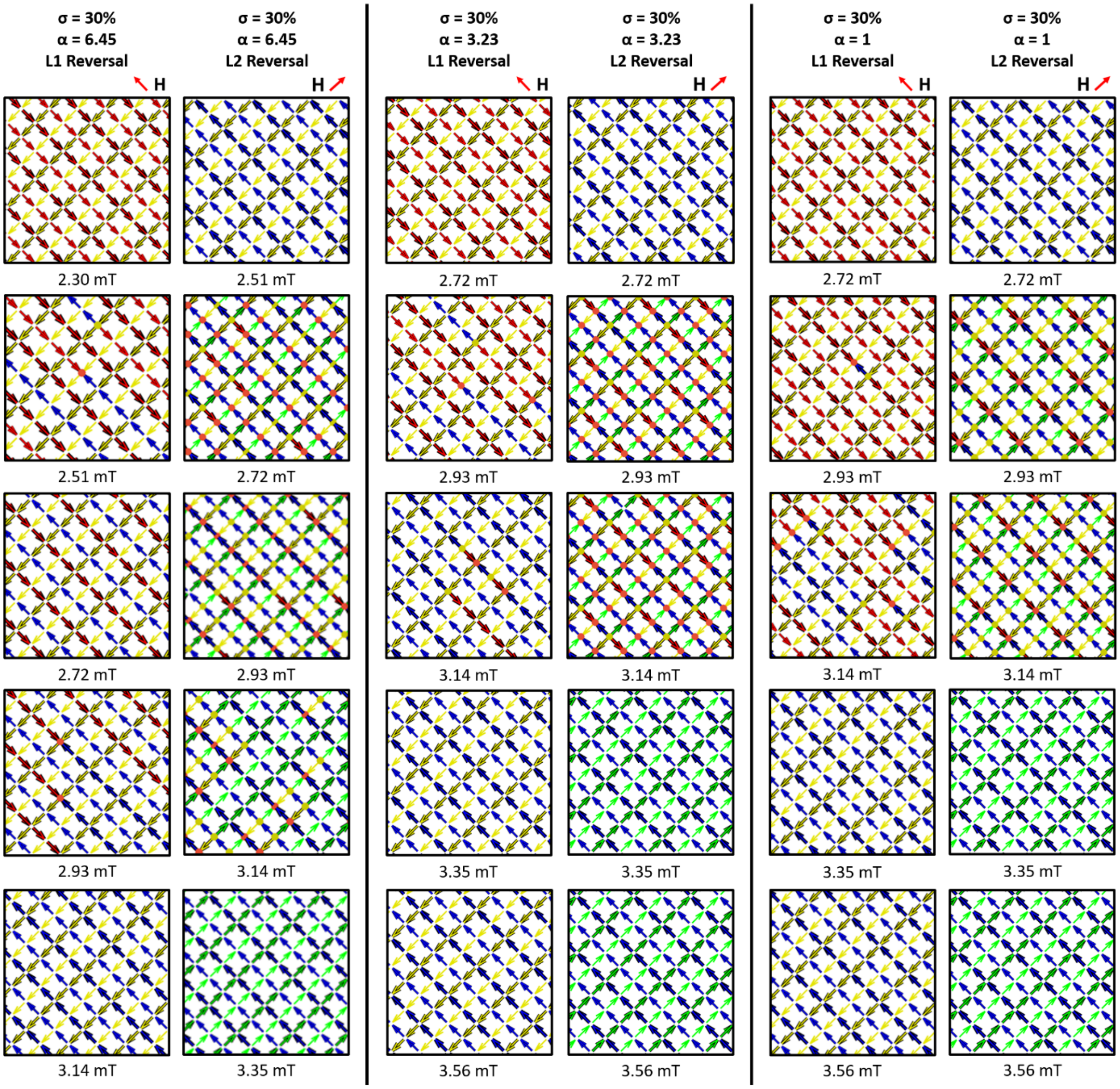


**Supplementary Fig. 12:** Full Monte-Carlo Simulations with varying surface energetics ($\alpha_{ij}$). Micromagnetic simulations indicate $\alpha_{ij}$ = 3.23, whereas these Monte-Carlo simulations most closely resemble the experimental results when $\alpha_{ij}$ = 6.45.
